# Effects of temperature, moisture, and metal salt content on dielectric properties of rice bran associated with radio frequency heating

**DOI:** 10.1038/s41598-018-22567-4

**Published:** 2018-03-13

**Authors:** Bo Ling, Xiaoli Liu, Lihui Zhang, Shaojin Wang

**Affiliations:** 1 College of Mechanical and Electronic Engineering, Northwest A&F University, Yangling, Shaanxi 712100 China; 2 College of Food Science and Engineering, Yangling, Shaanxi 712100 China; 3 Department of Biological Systems Engineering, Washington State University, Pullman, WA 99164-6120 USA

## Abstract

Dielectric heating including microwave (MW) and radio frequency (RF) energy has been regarded as alternative thermal treatments for food processing. To develop effective rice bran (RB) stabilization treatments based on RF and MW heating, dielectric properties (DPs) with dielectric constant (*ε*′) and loss factor (*ε*″) of RB samples at frequencies (10–3000 MHz), temperatures (25–100 °C), moisture content (MC, 10.36–24.69% w.b.) and three metal salt levels (0.05–2.00%) were determined by an open-ended coaxial probe and impedance analyzer. Results indicated that both *ε*′ and *ε*″ of RB samples increased with increasing temperature and MC. The increase rate was greater at higher temperature and moisture levels than at lower levels, especially at frequencies lower than 300 MHz. Cubic order models were developed to best fit the relationship between DPs of RB samples and temperature/MC at five frequencies with *R*^2^ greater than 0.994. Both *ε*″ and RF heating rate of RB samples increased significantly with added NaCl (2%), KCl (1%) and Na_6_O_18_P_6_ (2%). The obtained data are useful in developing computer models and simulating dielectric heating for RB stabilization and may also provide theoretical basis for synergistic stabilization of RB under combined dielectric heating with metal salts.

## Introduction

Rice bran (RB), a by-product of rice milling, is obtained from outer layer of the brown rice kernel accounting for 6–8% of the paddy rice^1^. The global paddy rice production was 741 million tons and this huge amount of production resulted in more than 50 million tons of RB in 2014^2^. RB is a nutrient-rich by-product mainly owing to its high contents of oils, fibers, proteins and phytochemicals. Although RB contains high nutritive constituents, very short shelf life due to lipid rancidity caused by lipase has limited the wide usage of RB as a food material^3^.

Several methods have been adopted to stabilize RB by reducing the activity of lipase. Among these methods, physical treatments using thermal energy with or without moistening are most commonly employed. However, hot air drying or steaming is the slow heating in large volume samples and may result in long treatment times and possible damage to RB quality. Novel thermal processing technologies, such as infrared (IR) and ohmic (OH) heating, have been also used for RB stabilization. Nevertheless, thinner sample thickness during IR heating commonly less than 1.0 cm may limit its use for mass production^4^. For OH heating, RB needs to be either unpackaged and in direct contact with the electrodes^5^. Therefore, it is important to explore an alternative stabilization method for achieving the high efficiency and maintaining the good RB quality.

Dielectric heating, including microwave (MW) and radio frequency (RF) energy, could rapidly raise the temperature of foodstuff volumetrically and significantly reduce the heating time^6,7^. Moreover, dielectric heating is more suitable for industrial scale treatments mainly due to deep penetrations^7^. To develop an effective dielectric heating treatment for food processing, it is important to understand dielectric properties (DPs), the major factors describing the interaction between electric fields and the food material. The DPs of foodstuffs commonly interested in most applications are dielectric constant (*ε*′) and loss factor (*ε*″), where the *ε*′ indicates the ability of food material to store energy and the *ε*″ reflects how well a food material absorbs energy from electric fields and convert electric energy to thermal energy. Moreover, the penetration depth calculated from *ε*′ and *ε*″ is an important parameter in characterizing temperature distributions and designing treatment bed thicknesses in dielectric heated foodstuffs. Therefore, knowledge of DPs of food materials is essential to the design, optimization and control of the thermal process with dielectric heating.

In the past 5 years, DPs have been studied over different frequency, temperature and moisture ranges of food and agricultural products for various purposes. Several studies have been conducted to investigate the DPs of flour materials from various agricultural products. For example, Boreddy and Subbiah^8^ reported that the DPs of egg white flour were maximum in the frequency range of 250–300 MHz at the temperature range for pasteurization. Ozturk *et al*.^9^ developed quadratic models for moisture contents (MCs), temperature and DPs of broccoli flour at two radio frequencies from 20 to 80 °C. Up to now, DPs of RB as influenced by temperature and MC and their heating behavior during RF treatments influenced by MC are still not available in the literature.

In addition, ionic components in foodstuff also have significant influence on their DPs. Nelson and Datta^10^ reported that the DPs of foodstuff are dependent on chemical composition and especially on the presence of the dissolved ions. For example, Wang *et al*.^11^ reported that addition of NaCl could raise both *ε*′ and *ε*″ of re-structured potato slices and significantly improved the MW freeze drying rate. Similarly, Lu *et al*. found that the NaCl content had a significantly positive effect on *ε*″ of meat batter, but also had a significantly negative effects on dielectric heating rate^12^. On the other hand, metal salts have been also reported as effective inhibitor for cereal lipase. For example, Munshi *et al*.^13^ found that lipid rancidity of RB could be inhibited by spraying solutions of FeCl_3_ or NiCl_2_ over the bran during storage. Doblado-Maldonado *et al*.^14^ reported that replaced tempering water by NaCl, KCl and FeNaEDTA solutions prior to wheat milling could reduce the lipid rancidity of whole wheat flour during storage. Based on the higher effectiveness of wet heating compared to dry heating for RB stabilization, metal salt solutions might be able to replace water to moistening the RB and exert the synergy effect for inactivation of lipase during RB stabilization using dielectric heating. Although there have been many studies reported the impact of salts on DPs of food materials, these studies are mainly focused on high MC (e.g., ≥50% w.b.) foods with added NaCl and those related to the influence of metal salts on DPs of low MC food materials, such as RB and its heating rate during RF treatment, are limited.

Therefore, the objectives of this study were: (1) to determine the DP data of RB from 10 to 3000 MHz at temperatures between 25 to 100 °C with four MCs, (2) to provide the empirical models describing RB’s DPs and penetration depth as a function of MC and temperatures at five interested frequencies, (3) to explore the influence of four metal salt solutions on the DPs and penetration depth of RB and (4) to evaluate the influence of MCs and metal salt solutions on the RF heating rate of RB.

## Results

### DPs of RB at different frequencies

**Figure 1:**
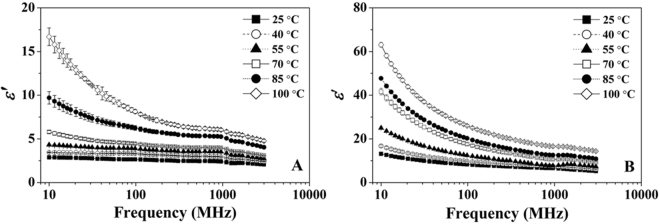
Frequency dependence of the dielectric constant of RB at 10.36% (**A**) and 24.69% w.b. (**B**) between temperatures range of 25 to 100 °C.

**Figure 2:**
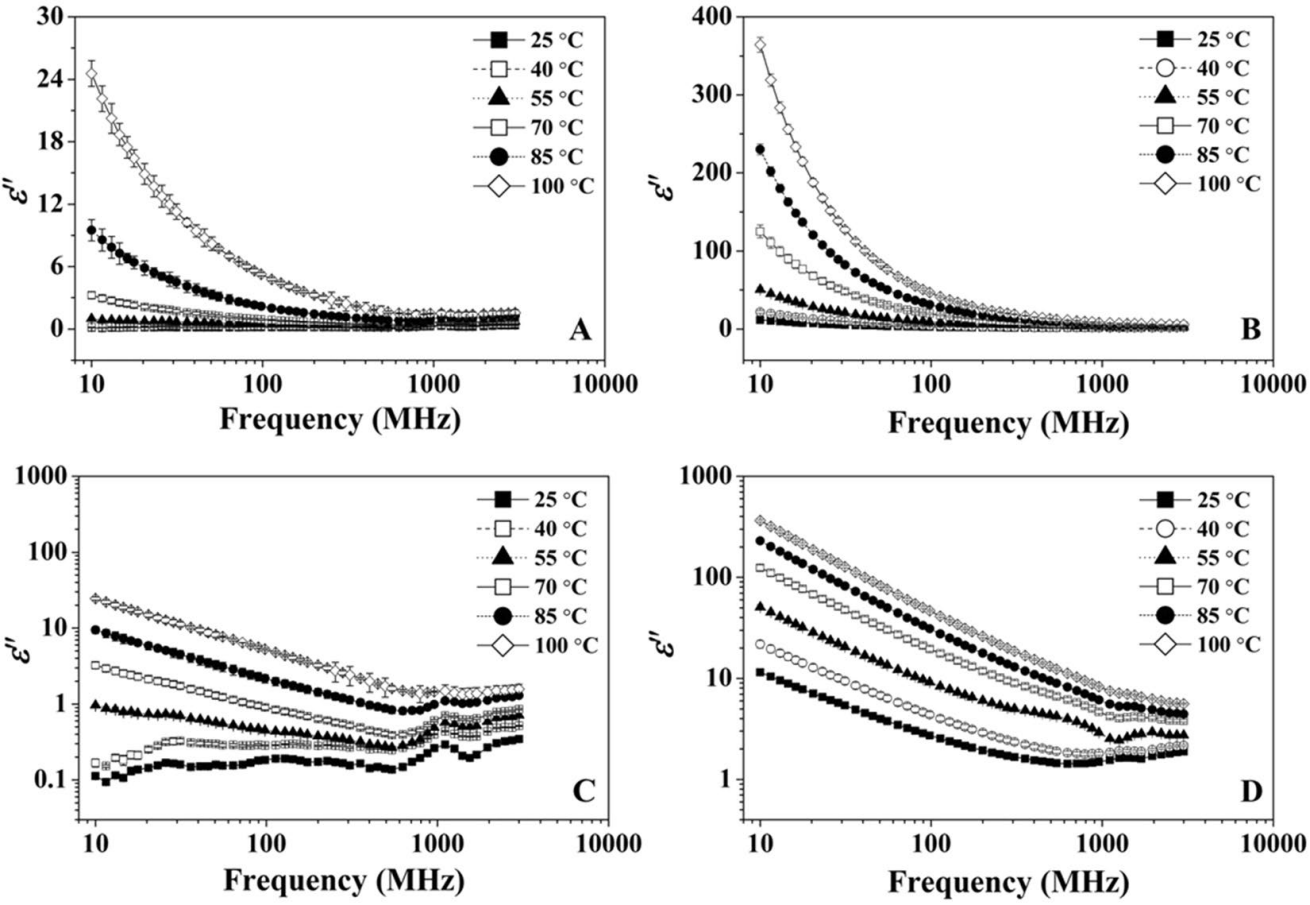
Frequency dependence of the dielectric loss factor of RB at 10.36% (**A**) and 24.69% w.b. (**B**) together with the log-log plot at 10.36% (**C**) and 24.69% w.b. (**D**) between temperatures range of 25 to 100 °C.

Figures 1 and 2 show the measured data of *ε*′ and *ε*″ of RB with MCs of 10.36 and 24.69% w.b. between 25 and 100 °C over the frequency range of 10 to 3000 MHz. Both *ε*′ and *ε*″ of two RB samples decreased with increasing frequency, especially at the frequencies lower than 300 MHz. The temperature had also significant effect on the frequency dependent DPs of RB. With the frequency increased from 13.56 to 2450 MHz, *ε*′ and *ε*″ of RB with 24.69% w.b. MC decreased from 12.22 to 5.48 and 9.55 to 1.81 at 25 °C (Figs 1B and 2B), respectively. However, the corresponding decreasing of *ε*′ and *ε*″ was from 54.37 to 14.90 and 283.77 to 5.75 at 100 °C, decreasing by 73 and 98%, respectively. In addition, the effect of frequency on DPs was more marked at the higher MC, for RB with MC of 24.69% w.b. and the *ε*′ and *ε*″ decreased from 22.42 and 40.6 to 7.62 and 2.74 over the frequency range of 13.56 to 2450 MHz at 55 °C (Figs 1B and 2B), decreasing by 66 and 93%, respectively. But for RB with MC of 10.36% w.b. the corresponding decreasing was from 4.27 and 0.85 to 2.84 and 0.67 (Figs 1A and 2A), decreasing by 33 and 21%, respectively. When the *ε*″ of RB with two MCs were plotted against frequency in the log-log plot (Figs 2C and 2D), it could be clearly seen that the negative linear relationships were obtained between *ε*″ and frequency over 10 to 300 MHz, especially for sample with MC of 24.69% w.b.

### Effect of temperature and MC on DPs of RB

**Figure 3:**
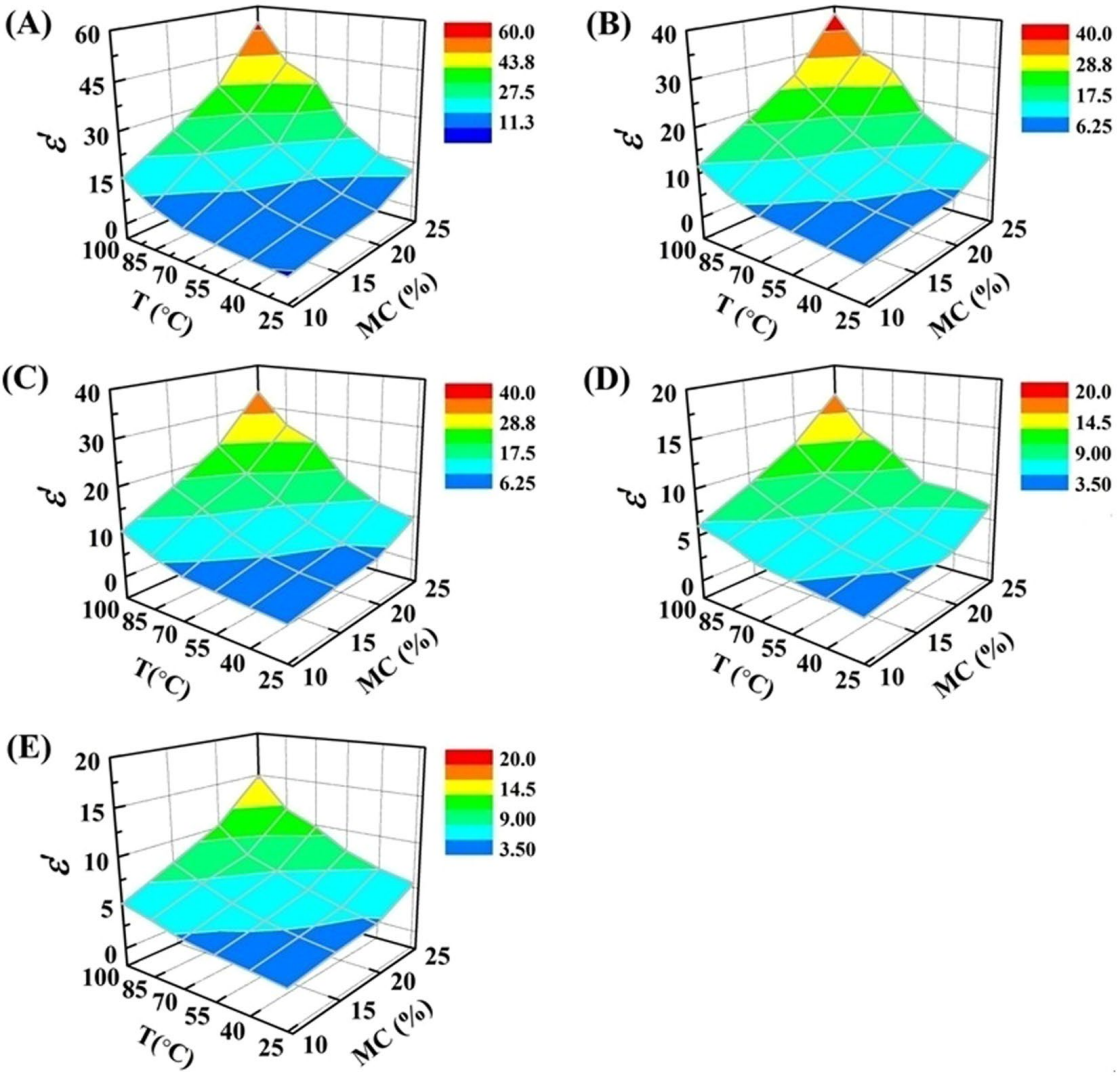
Dielectric constant of RB as function of MC and temperature at 13.56 (**A**), 27.12 (**B**), 40.68 (**C**), 915 MHz (**D**) and 2450 MHz (**E**) over a MC range from 10 to 25% w.b. and temperature range from 25 to 100 °C.

The temperature and MC dependency of the average *ε*′ of RB samples was shown in Fig. 3. It could be noted that *ε*′ of RB samples increased with increasing temperature and MC. For example, the *ε*′ of natural state RB (10.36% w.b.) increased from 2.78 to 11.46 with an increase in temperature from 25 to 100 °C at 27.12 MHz (Fig. 3B), whereas it increased from 2.14 to 4.99 at 2450 MHz (Fig. 3E). The rate of increase in *ε*′ with the increased temperature was rapid at samples with high MC, especially at radio frequencies range. Meanwhile, the *ε*′ increased slowly with increasing MC over MC range from 10 to 20% w.b., then increased rapidly to MC of 25% w.b. for radio frequencies range, especially at high temperatures. On the contrary, the *ε*′ increased gradually with increasing MC for all the temperatures at MW frequencies.

**Figure 4:**
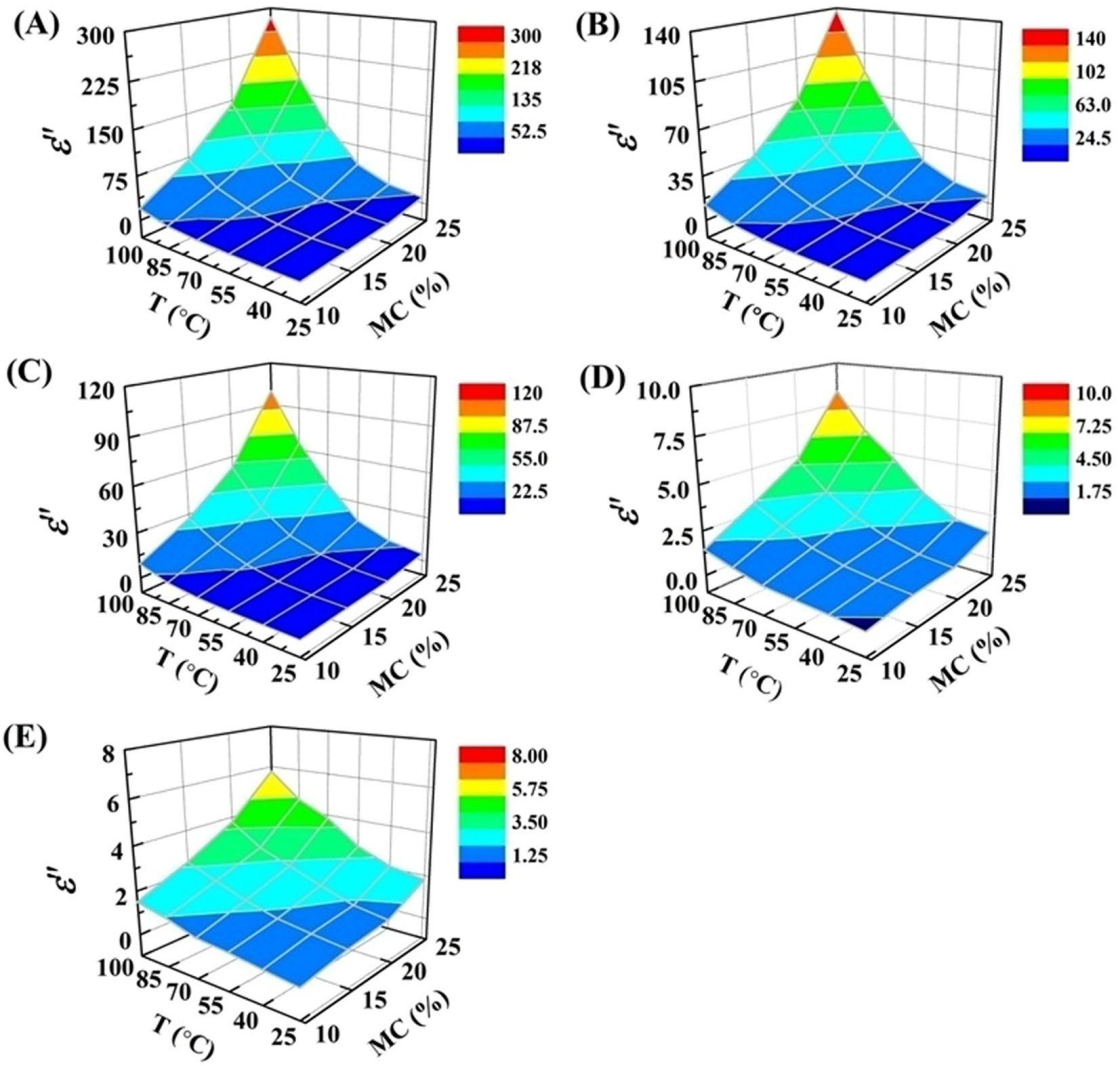
Dielectric loss factor of RB as function of MC and temperature at 13.56 (**A**), 27.12 (**B**), 40.68 (**C**), 915 MHz (**D**) and 2450 MHz (**E**) over a MC range from 10 to 25% w.b. and temperature range from 25 to 100 °C.

Figure 4 shows the *ε*″ of RB samples as a function of MC and temperature at five specific frequencies. Similar to *ε*′, the *ε*″ also increased with increases in either MC or temperature. The increase in *ε*″ of RB as a function of temperature was rapid at high MC levels compared to the low MC. As the temperature increased from 25 to 100 °C, the *ε*″ at 27.12 MHz increased from 1.3 to 71.5 at MC of 20.02% w.b. and from 0.2 to 12 at MC of 10.36% w.b., respectively (Fig. 4B). Moreover, the *ε*″ of RB increased rapidly with an increase in MC at high temperatures, especially for radio frequencies range.

### Regression analysis of the DPs of RB at five specific frequencies

Coefficients of the cubic order models with its significant terms describing the *ε*′ of RB as influenced by MC and temperature are listed in Table 1. The cubic order model as shown in the general form (Eq. 2) for each frequency was used to predict the *ε*′ of RB in the testing MC and temperature range. The terms *MC*, *T*, *MCT*, *MC*2 and *T*^2^ had strong influence on the model at all five frequencies (*p* < 0.0001). Each model provided a good fit to the *ε*′ at the significance level of 0.0001 and with *R*^2^ value greater than 0.994 for different frequencies. Table 2 shows the coefficients of the cubic order models with significant terms describing the *ε*″ of RB as influenced by MC and temperature. The linear (*MC* and *T*), interaction (*MCT* and *MC*^2^*T*) and squared (*MC*2 and *T*^2^) terms had strong influence on the model at all selected frequencies (*p* < 0.0001) except for three radio frequencies. The linear interaction (*MCT*2) term influenced strongly (*p* < 0.0001) the model at three radio frequencies only. The significant probability of each model was less than 0.0001 with *R*^2^ value above 0.995. Therefore, it can be concluded that the cubic order models are acceptable enough to predict both *ε*′ and *ε*″ of RB samples at any given MC and temperature at their respective frequencies. The DPs of foodstuffs increased with increasing MC and temperature and the temperature and MC dependency properties could change the electromagnetic power absorption, penetration depth and temperature distribution in foodstuffs^15^. Therefore, the models obtained from this study for predicting DPs of RB would be useful in computer model for simulating temperature histories of RB samples during dielectric heating and in designing the dielectric heating system employed with RF and MW frequencies.

**Table 1:** Regression coefficients of the cubic order models to predict the dielectric constant of RB as a function of MC (10–25% w.b.) and temperature (25–100 °C).

Regression coefficients in Eq. (3)	Regression coefficients at five selected frequencies				
	13.56 MHz	27.12 MHz	40.68 MHz	915 MHz	2450 MHz
*α*_0_	−23.15	−16.45	−14.84	−6.71	−8.86
*α*_1_	7.36^*1^	5.22^*^	4.50^*^	1.36^*^	1.90^*^
*α*_2_	−0.61^*^	−0.40^*^	−0.28^*^	0.14^*^	0.10^*^
*α*_12_	8.52 × 10^−3*^	9.06 × 10^−3*^	5.74 × 10^−3*^	−6.44 × 10^−3*^	−3.72 × 10^−3*^
*α*_11_	−0.50^*^	−0.35^*^	−0.30^*^	–0.08^*^	−0.12^*^
*α*_22_	7.14 × 10^−3*^	4.38 × 10^−3*^	3.07 × 10^−3*^	−1.79 × 10^−3*^	−1.50 × 10^−3*^
*α*_112_	4.91 × 10^−4^	2.21 × 10^−4^	1.90 × 10^−4^	1.43 × 10^−5^	7.98 × 10^−5^
*α*_122_	1.91 × 10^−5^	1.18 × 10^−5^	2.58 × 10^−5^	9.15 × 10^−5^	5.23 × 10^−5^
*α*_111_	0.01	7.30 × 10^−3^	6.24 × 10^−3^	1.83 × 10^−3^	2.50 × 10^−3^
*α*_222_	−2.25 × 10^−5^	−1.30 × 10^−5^	−8.94 × 10^−6^	5.91 × 10^−6^	7.15 × 10^−6^
*Model*	*	*	*	*	*
*R* ^2^	0.994	0.995	0.996	0.995	0.998

^1^*Indicated model terms significantly influence the model at 0.0001 probability levels.

**Table 2:** Regression coefficients of the cubic order models to predict the dielectric loss factor of RB as a function of MC (10–25% w.b.) and temperature (25–100 °C).

Regression coefficients in Eq. (3)	Regression coefficients at five selected frequencies				
	13.56 MHz	27.12 MHz	40.68 MHz	915 MHz	2450 MHz
*α*_0_	−347.50	−161.92	−113.67	−8.88	−3.51
*α*_1_	53.44	25.82	18.49	1.78^*^	0.75^*^
*α*_2_	7.02^*1^	2.99^*^	1.99^*^	0.04^*^	5.35 × 10^−3*^
*α*_12_	−0.64^*^	−0.29^*^	−0.20^*^	−8.05 × 10^−3*^	−1.92 × 10^−3*^
*α*_11_	−2.64^*^	−1.31^*^	−0.95^*^	−0.10^*^	–0.05^*^
*α*_22_	−0.07^*^	−0.03^*^	−0.02^*^	−2.02 × 10^−4*^	7.20 × 10^−5*^
*α*_112_	0.01^*^	5.77 × 10^−3*^	4.05 × 10^−3*^	2.24 × 10^−4*^	6.63 × 10^−5^
*α*_122_	3.40 × 10^−3*^	1.52 × 10^−3*^	1.05 × 10^−3*^	4.25 × 10^−5^	1.71 × 10^−5^
*α*_111_	0.04	0.02	0.02	1.95 × 10^−3^	9.47 × 10^−4^
*α*_222_	2.00 × 10^−4^	8.33 × 10^−5^	5.49 × 10^−5^	8.92 × 10^−8^	−5.83 × 10^−7^
*Model*	*	*	*	*	*
*R* ^2^	0.998	0.995	0.998	0.996	0.996

^1^*Indicated that model terms significantly influence the model at 0.0001 probability levels.

### Effect of temperature and MC on penetration depth of RB

**Figure 5:**
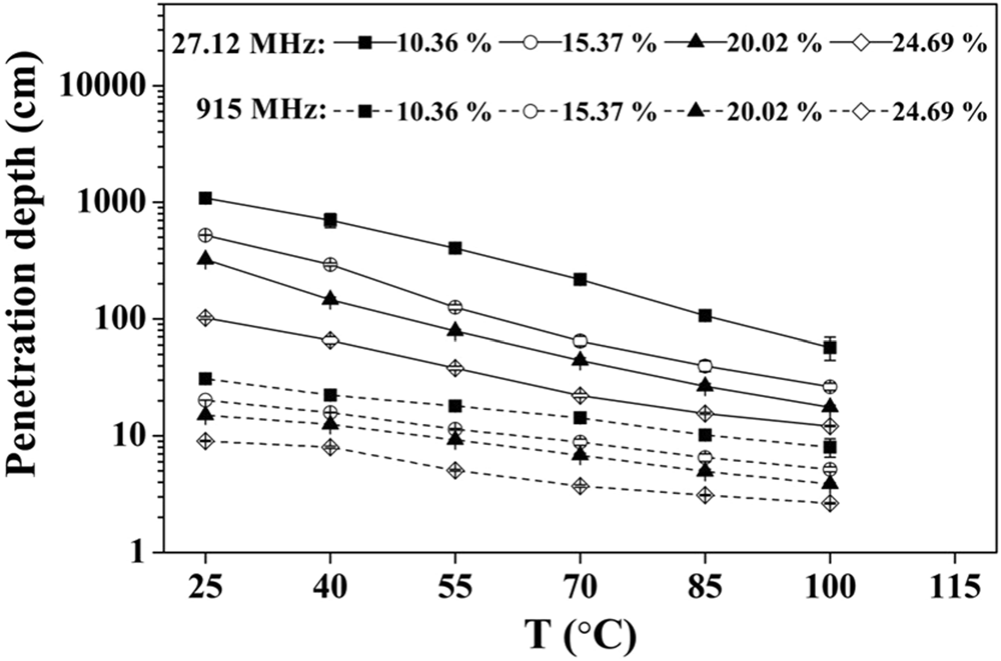
MC and temperature dependent penetration depths of RB at 27.12 and 915 MHz.

Figure 5 shows the calculated power penetration depth of electromagnetic energy in RB samples with four MCs at two typical frequencies (27 and 915 MHz) commonly used for industrial dielectric heating treatments. As shown in Fig. 5, the penetration depth at 27.12 MHz was many times (from 5 to 35) larger than that at 915 MHz depending on the MC and temperature of RB samples. For example, penetration depth at 27.12 MHz ranged from 1086 to 12 cm for RB sample with MC between 10 to 25% w.b. over temperature range of 25 to 100 °C, but the corresponding values at 915 MHz were ranged from 31 to 3 cm. Moreover, the penetration depth decreased with an increase in MC and temperature at two selected frequencies. For example, the penetration depth of natural state RB (10.36% w.b.) decreased from 1086 and 30.89 to 57.02 and 8.99 cm when the temperature increased from 25 to 100 °C at 27.12 and 915 MHz, respectively. However, the corresponding penetration depth decreased from 101 and 9 cm to 12 and 3 cm when the RB was moistening to 24.69% w.b.

### Effect of different metal salts on DPs and penetration depth of RB

**Figure 6:**
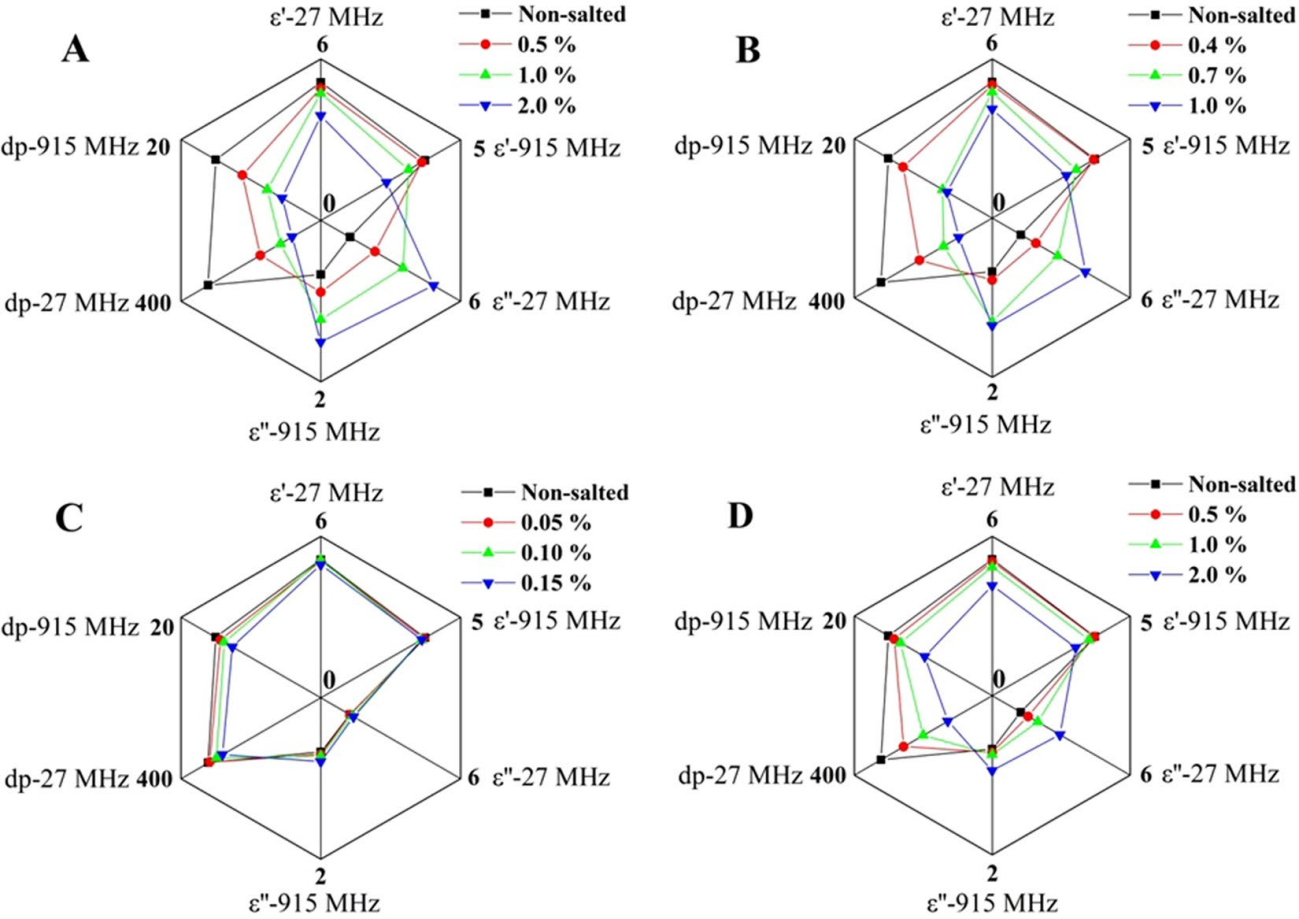
Effect of added NaCl (**A**), KCl (**B**), FeNaEDTA (**C**) and Na_6_O_18_P_6_ (**D**) on the DPs and penetration depth of RB (20% w.b.) at 27.12 and 915 MHz at 25 °C.

Figure 6 shows that the four metal salts with different levels influenced DPs and penetration depth of RB samples with MC of 20% w.b. at two typical frequencies and 25 °C. It could be noted that added NaCl, KCl and Na_6_O_18_P_6_ all appeared to exert a depressing effect on *ε*′ of RB, especially at higher levels (2% for NaCl and Na_6_O_18_P_6_ and 1% for KCl) (*p* < 0.05). However, added metal salts had a significantly (*p* < 0.05) positive effect on *ε*″, except for FeNaEDTA within the selected levels. Both the absolute increase and the relative increase were larger at 27.12 MHz than at 915 MHz for all three metal salts. For example, at the frequency of 27.12 MHz, the absolute increases of *ε*″ between non-salted and salted samples with the highest level were 3.59, 2.80 and 1.70 for NaCl (2%), KCl (1%) and Na_6_O_18_P_6_ (2%), respectively, representing a relative increase of 287%, 224% and 136%. While at the frequency of 915 MHz, the corresponding absolute and relative increases were 0.84, 0.68, 0.27 and 125%, 101%, 40%, respectively.

Metal salts also had a negative effect on penetration depth of RB and there was an inverse relationship between *ε*″ and penetration depth. As *ε*″ increased with increasing salt levels, the penetration depth decreased significantly. This effect was marked both at 27 MHz than 915 MHz. For example, with NaCl increased from 0 to 2%, the penetration depth decreased from 322 to 82 cm at 27.12 MHz, while the corresponding decrease was from 15 to 5.6 cm at 915 MHz. These results indicate that the metal salt content instead of MC should be carefully monitored during RB stabilization using combined RF heating with metal salt solutions.

### Effect of moisture and salt content on RF heating rate of RB

**Figure 7:**
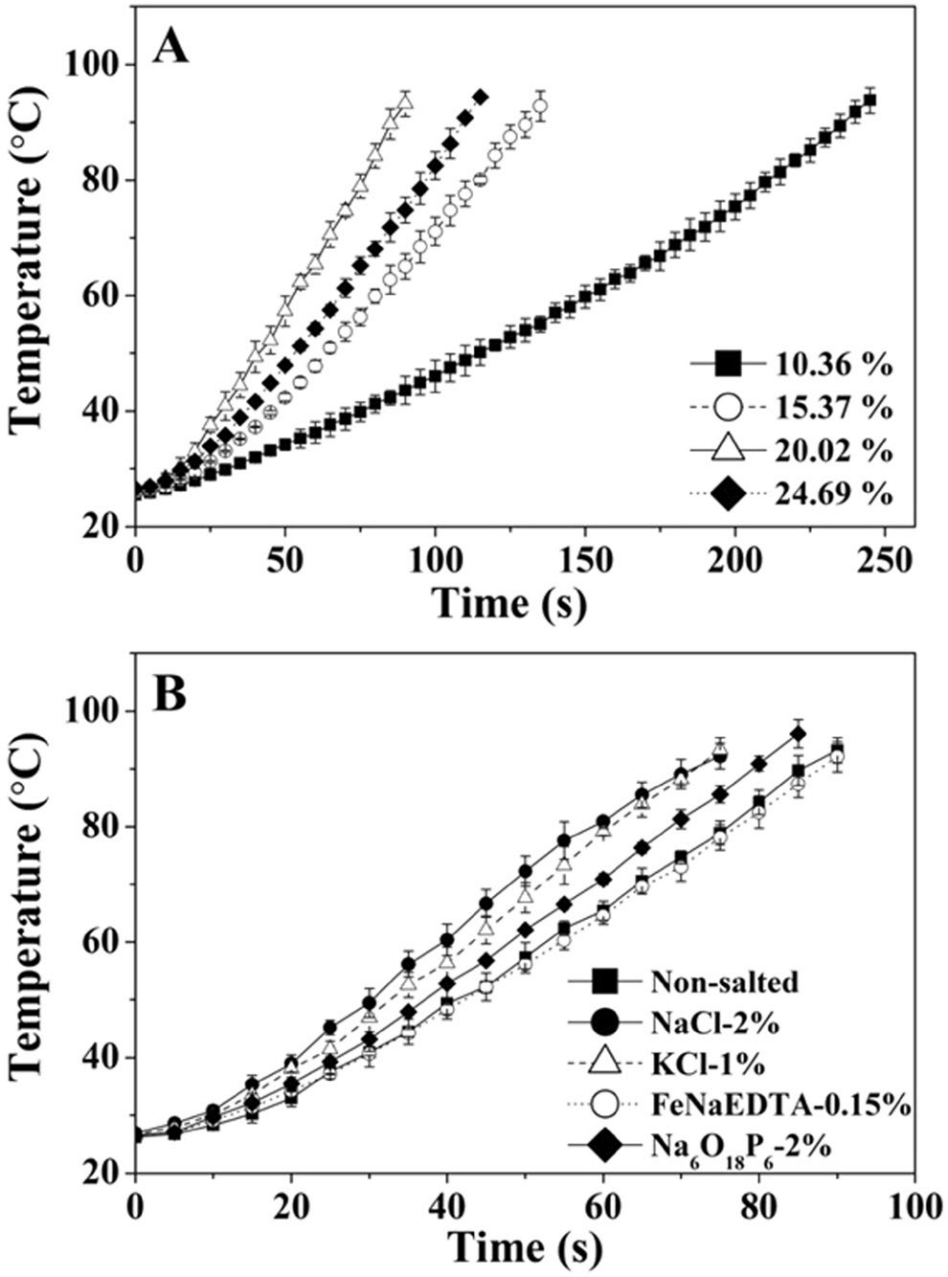
Temperature-time histories of RB samples with different moisture (**A**) and metal salt contents (20% w.b.) (**B**) during RF heating.

Figure 7A shows the average temperature-time histories at the center of 200 g RB samples with different MCs when heated in a 6 kW 27.12 MHz RF system at 10 cm electrode gap. About 240, 135, 90 and 110 s were needed to heat 200 g RB samples from 25 to 90 °C and the average heating rates were 0.27, 0.47, 0.71 and 0.58 °C/s for MCs of 10.36, 15.37, 20.02 and 24.69% w.b., respectively. It could be also noted that the RF heating rate increased significantly (*p* < 0.05) with increasing MC up to 20.02% w.b., then followed the significant (*p* < 0.05) decrease when the MC reached 24.69% w.b. To further study the influence of metal salt on RF heating rates, average temperature-time histories of the RB sample (20% w.b.) with highest metal salt levels were obtained and are shown in Fig. 7B. The heating rates were determined as 0.71, 0.85, 0.84, 0.70 and 0.80 °C/s for not-salted, NaCl (2%), KCl (1%), FeNaEDTA (0.15%) and Na_6_O_18_P_6_ (2%) samples, respectively. The added NaCl, KCl and Na_6_O_18_P_6_ could further improve RF heating rate significantly (*p* < 0.05), while FeNaEDTA (0.15%) had no significant effect (*p* > 0.05).

## Discussion

At the natural state of RB (10.36% w.b.), both *ε*′ and *ε*″ were less than 3.0 at 25 °C (Figs 1A and 2A), which was less than the mean DP values (*ε*′ = 3.01 and *ε*″ = 4.15) of rice flour (12% w.b.) over the frequency range of 500 to 2500 MHz reported by Ahmed *et al*.^16^. This may be mainly due to the different composition between RB and rice flour. Ryynänen^17^ reported that the loss mechanism of foodstuffs with high MC was mainly dependent on the ionic conduction and dipole relaxation at frequencies lower than 300 MHz. In the natural state of RB (10.36% w.b.), water molecules are mainly bound to carbohydrates and proteins and the solvent effect of ions was weaker, resulting in low *ε*″ values in the low frequency range (Fig. 2C). The negative linear relationship between *ε*″ and frequency in the log-log plot (Figs 2C and 2D) could be because the ionic conduction played the dominant role for loss mechanism at frequencies lower than 300 MHz and the dipole polarization was the main loss mechanism at MW frequencies. Based on a modified Debye equation analytical method, Liu *et al*.^18^ found that the ionic conduction played a major role for loss mechanisms of bread, while the dipole relaxation was very small at low frequencies and moderate at high frequencies. Similar relationships were also found in nut kernel, vegetable and fruit flour with high MC^19–21^.

The DPs of foodstuffs mainly depend on free water content, as the MC increases with increasing free water content in the RB and the increases of temperature result in increases of the ionic mobility. Thus the increase in *ε*′ and *ε*″ with an increase in the MC and temperature might be due to the increased polarization of molecules and ionic conductivity^22^. Food materials can store and dissipate electric energy when placed in an electromagnetic field and the *ε*″ values determine the ability of electric energy absorption, resulting in temperature changes^23^. MC is a critical factor in dielectric heating treatment and also important for enzyme inactivation due to increase in the conformation flexibility of the enzyme^24^. As the MC and temperature increases result in increases in the free water content and its mobility in RB and thereby increases of the *ε*″ and conformation flexibility of the enzymes in RB. Therefore, more electromagnetic energy could be absorbed by RB samples with higher MC and the higher heating rate could be obtained with easy inactivation enzymes during RB stabilization. From the basic scientific viewpoint, it would suggest that pre-moisturization treatment was an effective method to improve the efficiency of RB stabilization by dielectric heating.

The smaller penetration depth values at 915 MHz result in more surface heating, indicating that RF was more suitable for industrial scale RB stabilization over the MW treatment when the better heating uniformity is considered as a major goal. To obtain a better heating uniformity and effective stabilization of RB, the thickness of RB should not be larger than penetration depth of the dielectric heating system. Therefore, the penetration depth values shown in Fig. 5 could be used as practical guidance for designing the thickness of RB samples during stabilization by RF and MW heating with or without moistening.

Added metal salts leads to *ε*′ gradually decreased while *ε*″ increased significantly (*p* < 0.05) at similar conditions. Ryynänen^17^ reported that the *ε*′ of water was not affected by salt content at low levels, but higher salt content would reduce *ε*′ and this may be because the dissolved ions from salt could bind the free water molecules and the degree of binding depends on the size and charge of the ions, which results in water polarization and consequently decreases in *ε*′^25^. Based on the determination of water activity (*a*_w_) in our study, the average *a*_w_ values determined by an AquaLab *a*_w_ meter (Model 4TE, Decagon Devices, Inc., Pullman, WA, USA) were 0.904, 0.858, 0.864, 0.902 and 0.869 for samples with non-salted, 2% NaCl, 1% KCl, 0.15% FeNaEDTA and 2% Na_6_O_18_P_6_ at 25 °C, respectively. These results could be further proved that added metal salts could reduce free water content in RB, thus, decrease in the *ε*′. However, for the FeNaEDTA, it is probable due to its low added levels and chelated form of the ferric, the number of dissolved sodium (Na^+^) and ferric (Fe^3+^) ions were too low to alert the DPs of the RB. The significant increases in *ε*″ of food materials after added salt were also observed in meat batter and butter reported by Ahmed *et al*.^26^ and Lu *et al*.^12^. They generally considered that dissolved salts caused the *ε*″ increase to above that of pure water due to addition of conductive charge carriers related to the size and charge of the dissolved ions.

Many factors, such as chemical composition of material, frequency of electromagnetic wave, temperature, all influence the related DPs and the resultant dielectric heating behavior. When the RB samples were heated in a RF system, the electromagnetic energy was absorbed by the RB and then converted to the thermal energy (*P*) that can be calculated by the equation^27^:

1 $$P=2\pi f{V}^{2}{\varepsilon }_{0}\varepsilon ^{\prime\prime} $$

where *f* and *V* are the frequency (Hz) and electric field strength (V/m) with *ε*_0_ of vacuum (8.85 × 10^−12^ F/m). Thus, based on the above equation, for a given RF system and fixed electrode gap, the portion of electromagnetic energy transferred to thermal energy depends on the *ε*″ of the RB, which mainly depends on MC and metal salt in our study^23^. The natural state RB (10.36% w.b.) with lowest *ε*″ resulted in the lowest RF heating rate, indicating that it takes long time to achieve the thermal inactivation temperature of lipase during stabilization and its quality may be damaged during long time heating. On the contrary, RB samples with higher MC resulting in higher *ε*″ would absorb more electromagnetic energy under same RF heating time, thus causing a higher heating rate. However, when the *ε*″ was higher than 1.25 at 25 °C for RB sample with MC of 20.02% w.b, the heating rate had no longer increase. Similar results were also found in RF treated peanut kernels and red pepper powders^28,29^, in which the increasing MC of materials resulted in the increased loss factor, causing RF heating rates increased initially and then decreased. Wang *et al*.^11^ reported that added NaCl could raise the MW freeze drying rate of re-structured potato slices significantly. This was explained by phenomenon, in which the added salt resulted in increases of the *ε*″ of the potato, thus improving energy absorption ability of foodstuffs.

Many studies have shown that the *ε*″ must be within a reasonable range to obtain an efficient RF heating for various food and agricultural products. For example, Birla *et al*.^30^ found that the maximum heating rate was obtained when the *ε*″ of model fruit (prepared from 1% gellan gel) reached 180 between the studied range of 80 and 350. Jiao *et al*.^27^ also suggested that for the peanut butter, the maximum RF heating rate would occur when the values of *ε*′ and *ε*″ were comparable. In this study, the maximum RF heating rate was obtained when the MC of the RB was 20.02% w.b. with added 2% NaCl or 1% KCl. This characteristics of salted RB samples may lead to pre-heating within a reasonably short time by RF energy to achieve the target inactivation temperature and subsequently holding it in hot air to complete the effective stabilization.

## Conclusion

The DPs of RB was greatly influenced by frequency, MC and temperature. Both *ε*′ and *ε*″ of RB samples decreased with increasing frequency or decreasing temperature and MC. The MC and temperature dependence of the DPs of RB at five specific frequencies could be described by cubic order model and each model provided a good fit to the DPs of RB at the significance level of 0.0001 with coefficient of the determination *R*^2^ value greater than 0.994. The RF heating rate of the RB samples was dependent on MC up to 20.02% (w.b.), but there was a significant decrease above this level. Added NaCl (2%), KCl (1%) and Na_6_O_18_P_6_ (2%) could increase in *ε*″ and RF heating rate and decrease in *d*_p_ of RB sample with MC of 20% (w.b.) significantly. The larger *d*_p_ at RF frequencies indicated that RF heating was suitable for industrial scale RB stabilization and pre-moisturization or salted treatment by metal salt solutions could further improve the efficiency of RB stabilization by RF heating.

## Materials and Methods

### Materials

RB obtained from the paddy variety of ‘Shanyou63’ (indica type), which represents a general variety grown in China, was used in the study. Freshly milled RB was directly collected from the milling unit in a local milling plant, after sieving the bran with a British Standard sieve no. 40 to remove broken grains, sand and other foreign materials. The RB was sealed into polyethylene bags at 5 °C until testing. NaCl, KCl, FeNaEDTA and Na_6_O_18_P_6_ (Analytical reagent) were purchased from Sinopharm Chemical Reagent Co., Ltd (Shanghai, China). The chemical compositions of the RB were determined with AOAC^31^ standard methods and are summarized in Table 3.

**Table 3:** Chemical compositions (Ave ± SD over three replicates) of studied RB.

Compositions	Content (g/100 g w.b.)	Methods
Protein^a^	13.24 ± 0.25	AOAC 950.48
Fat	22.01 ± 0.93	AOAC 948.22
Moisture	10.36 ± 0.08	AOAC 925.40
Ash	11.67 ± 0.06	AOAC 950.49
Carbohydrate	42.71	Estimated by difference^b^

^a^Protein was calculated based on nitrogen conversion factor of 5.95.

^b^Carbohydrate content = 100% − (% moisture + % protein + % fat + % ash).

### Preparation of sample with different MCs

MC of raw RB was commonly between 10–15% w.b. and according to the previous studies for RB stabilization with moisture addition^32,33^. RB samples with MC of 10–25% w.b. were selected in this study, higher MC was not considered since RB samples were easy to be agglomerated. RB with the original MC of 10.36% w.b. was adjusted to 15.37, 20.01 and 24.69% w.b. by sprayed the pre-calculated quantity of deionized water and then hydrated samples were stored in zipper-lock bags for 48 h at 5 °C and mixed by hand twice in a day for further moisture equilibrium.

### Preparation of sample with different salt contents

Because our intent was to explore the influence of metal salts that have inhibitory effect on cereal grain lipase on DPs of RB, the desired level of each metal salt was selected from literature based on the enzyme inactivation and acceptable levels in food processing. Salted samples were prepared by spraying pre-calculated quantity of metal salt solutions with different concentrations to the RB at the initial MC of 10.36% w.b. and the final MC (20% w.b.). Then they were stored in zipper-lock bags for 48 h at 5 °C and mixed by hand twice in a day for further equilibrium. Detail methods for prepared salted samples are summarized in Table 4.

**Table 4:** Preparation of metal salt solutions for conditioning using 100 g of RB samples with MC of 10.36% w.b.

RB/treatment	Desired salt concentration (g/100 g flour 10.36% w.b.)	Desired MC of RB (% w.b.)	Water/salt solution needed (mL)	Concentration of salt solution (g/mL)	Ref.
NaCl	0.50	20	12.05	0.041	^14,34^
	1.00			0.083	
	2.00			0.166	
KCl	0.40			0.033	^14,35^
	0.70			0.058	
	1.00			0.083	
EDTAFeNa	0.05			0.004	^13,34^
	0.10			0.008	
	0.15			0.012	
Na_6_O_18_P_6_	0.50			0.041	^36^
	1.00			0.083	
	2.00			0.166	

### Determination of tapped density

Since DPs are density dependent, any small vibration would change the density of flour material during transportation and processing. Various vibrations may cause RB to a nearly constant density termed as tapped density before stabilization. Therefore, RB samples with different MC were made at tapped density before determining its DPs. Based on the method of Bansal *et al*.^37^, 10 g RB was carefully placed in a graduated glass cylinder (25 mL with 1.77 cm in inner diameter), then the cylinder was tapped on table for 15 times from a height of 3 cm and the tapped volume was recorded for calculated tapped density of RB. The determined tapped density of RB is summarized in Table 5.

**Table 5:** The tapped densities (Ave ± SD over three replicates) of RB at four MC levels.

MC (%w.b.)	Tapped density (g/cm^3^)
10.36	0.364 ± 0.003
15.37	0.407 ± 0.006
20.02	0.453 ± 0.007
24.69	0.528 ± 0.006

### Determination of dielectric properties

**Figure 8:**
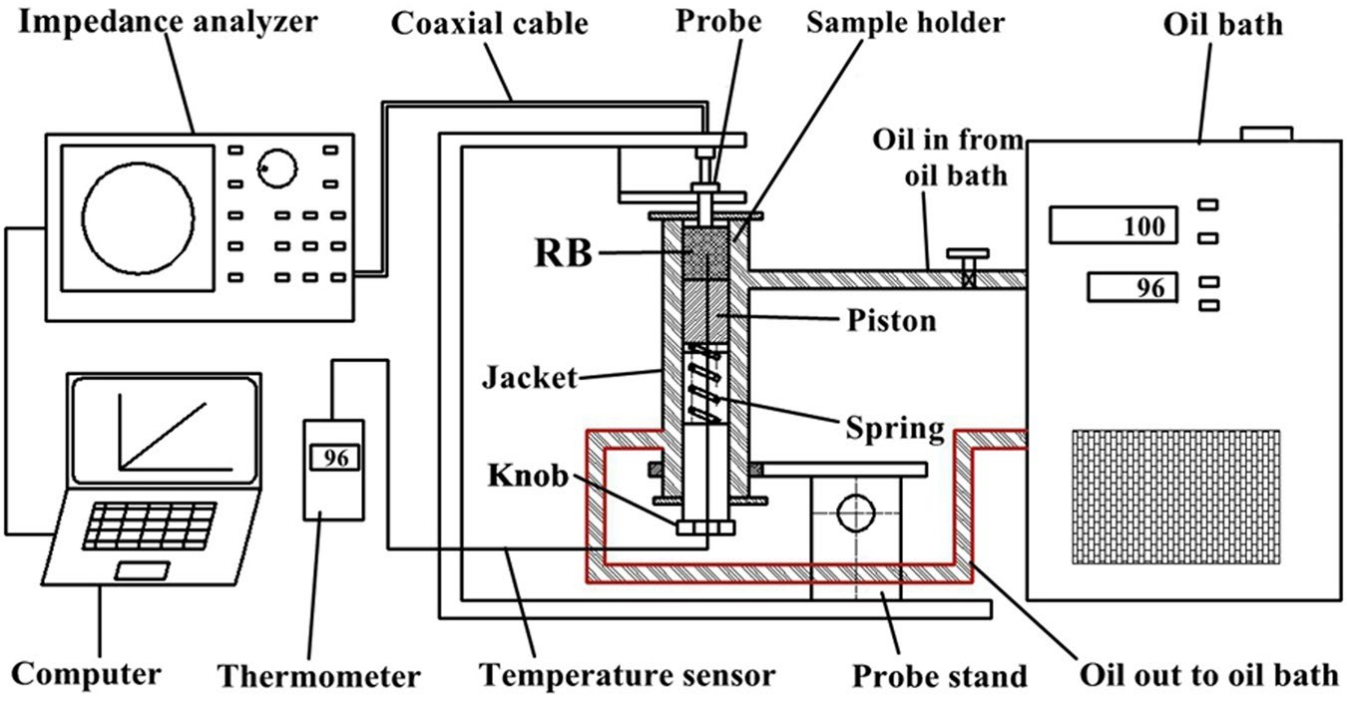
Schematic diagram of the dielectric property measurement system.

The DPs of RB samples were determined by an open-ended coaxial probe system (Fig. 8) which is mainly consisted of an E4991B-300 impedance analyzer, a 85070E-020 open-ended coaxial probe, a computer with 85070E dielectric probe kit software, an E4991B-10 calibration kit (Keysight Technologies Co., Ltd., Palo Alto, California, USA), a custom-built sample test holder (23 mm in inner diameter) and a SST-20 oil circulated bath (Guanya Constant Temperature Cooling Technology Co. LTD., Wuxi, China). The measurement frequency range for the analyzer was 1 to 3000 MHz and the sample holder was an oil-jacketed cylindrical holder made up of two coaxial stainless steel tubes and was connected to oil circulated bath for cyclic heating the samples. A pre-calibrated type-T thermocouple (TMQSS- 020U-6, Omega Engineering, Inc., Stamford, CT, USA), which was inserted into the center of the holder, was used to monitor the sample temperature. The high temperature probe and a rotating knob were connected to the top and bottom of the sample holder, respectively. The sample holder was airtight to maintain the sample MC during the measurement and its volume can be adjusted by rotating the knob to a certain position.

RB was taken from the refrigerator and placed at ambient conditions (25 °C) for 12 h to equilibrate. Before determining the DPs, the impedance analyzer was turned on and warmed up for 0.5 h and then calibrated following the standard procedure described by Bedane *et al*.^38^. Once the system was calibrated, known weights of RB calculated form tapped density determined in the previous section were put into sample holder and gently compressed by rotation of the knob to a specific position to make the density matched with the tapped density of RB at the same MC. The sample temperature was controlled by circulating oil from an oil bath into the jacket and raised from 25 to 100 °C in 15 °C increments. This temperature range was selected based on thermal inactivation studies of lipase in RB reported by Brunschwiler *et al*.^39^. After the sample temperature achieved the set value, measurements of the DPs data were conducted at 51 frequencies with a log sweep from 10 to 3000 MHz, which include three radio frequencies (13.56, 27.12 and 40.68 MHz) and two MW frequencies (915 and 2450 MHz) allocated by US Federal Communication Commission (FCC) for industrial applications. Three replicates were conducted at each MC level and the mean values were reported.

### Determination of penetration depth

The penetration depth of electric energy in a given material is defined as the distance in meters where the electric energy is reduced to 1/*e* of the energy passing the material surface and can be calculated by the following equation^40^:

2 $${d}_{p}=\frac{c}{2\pi f\sqrt{2\varepsilon \text{'}[\sqrt{1+(\frac{\varepsilon ^{\prime\prime} }{\varepsilon ^{\prime} }){}^{2}}-1]}}$$

where *c* is the velocity of light in vacuum (3 × 10^8^ m/s).

### Temperature profile of RB during RF heating

**Figure 9:**
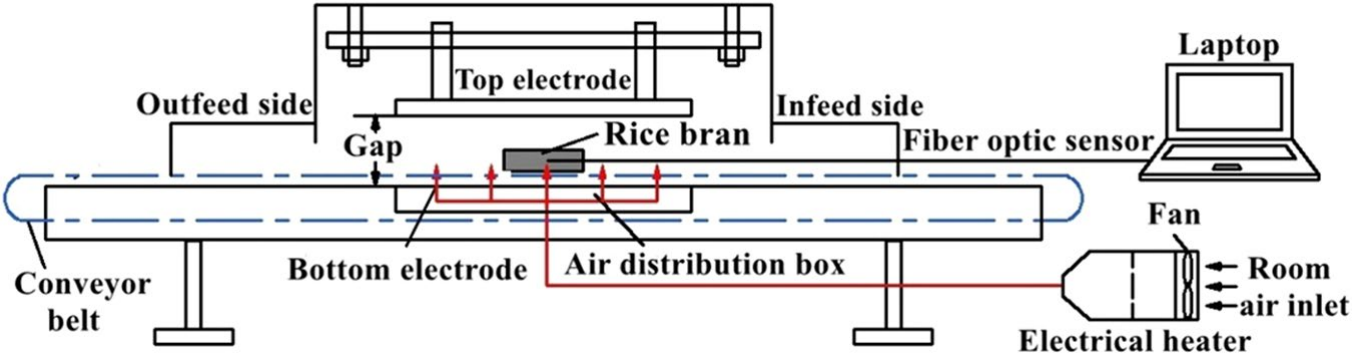
Schematic view of the pilot-scale 6 kW, 27.12 MHz RF system^41^.

RF heating was conducted in a 6 kW, 27.12 MHz pilot-scale free running oscillator RF system (SO6B, Strayfield International, Wokingham, U.K.) (Fig. 9). Each batch of 200 g non-salted and salted RB samples (~25 °C) was spread uniformly in a cylindrical polypropylene container (12 cm in diameter and 5.5 cm in depth) with 5 cm thickness. The container filled with RB was placed on the centre of the bottom electrode and the electrode gap was maintained at 10 cm in this study to obtain a faster heating rate without arcing. The RF system was turned off until the geometry center (cold spot) of RB achieved the 90 °C. A fiber-optic temperature sensor (HQ-FTS- D120, Heqi Technologies Inc., Xian, China) connected to a data logger (FTS-P104, Heqi Technologies Inc., Xian, China) inserted at the geometry center of RB sample was used to record the sample temperature profile during RF heating.

### Model analysis

To study the MC and temperature dependent DPs of RB, the correlations of MC and temperature (T, °C) with DPs of RB at three radio frequencies and two MW frequencies were determined by response surface method (RSM). The model fitting and regression analysis were performed using Design- expert 8.0.6 (Stat-Ease, Inc. Minneapolis, MN, USA). The general form of the fitted cubic order model was as follows:

3 $$\begin{array}{ccc}\varepsilon ^{\prime} \,{\rm{o}}{\rm{r}}\,\varepsilon ^{\prime\prime}  & = & {\alpha }_{0}+{\alpha }_{1}MC+{\alpha }_{2}T+{\alpha }_{12}MCT+{\alpha }_{11}M{C}^{2}+{\alpha }_{22}{T}^{2}\\  &  & +{\alpha }_{112}M{C}^{2}T+{\alpha }_{122}MC{T}^{2}+{\alpha }_{111}M{C}^{3}+{\alpha }_{222}{T}^{3}\end{array}$$

where, *α*_0_, *α*_1_, *α*_2_, *α*_12_, *α*_11_, *α*_22_, *α*_112_, *α*_122_, *α*_111_ and *α*_222_ are regression coefficients. The significant terms, significance of the model and coefficient of the determination (*R*^2^) ability of the model to fit the experimental data were automatically given by the software.

### Statistical analysis

Significant differences (*p* < 0.05) between different data were analyzed using variance and Tukey’s honestly significant difference (HSD) test in the statistical software SPSS 16.0 version (SPSS Inc., Chicago, IL, USA).
